# Inpatient Management and Treatment of a Giant Pancreatic Pseudocyst: A Case Report

**DOI:** 10.7759/cureus.19990

**Published:** 2021-11-29

**Authors:** William R Billari, Dwyer Roche, Jeremy V DiGennaro, Michael J Shallcross

**Affiliations:** 1 Radiology, Allegheny Health Network, Pittsburgh, USA; 2 Osteopathic Medicine, Edward Via Virginia College of Osteopathic Medicine, Blacksburg, USA; 3 Internal Medicine, Riverside Regional Medical Center, Newport News, USA

**Keywords:** pancreatitis complications, giant pancreatic pseudocyst, interventional gastroenterology, pseudocyst drainage, pseudocyst of the pancreas

## Abstract

Pancreatic pseudocyst formation is a common sequela of pancreatitis caused by alcohol use or gallstones. Giant pancreatic pseudocyst is an infrequently reported but serious complication of pancreatitis. Due to the large volume of pancreatic fluid containing active enzymes, giant pancreatic pseudocysts may require surgical intervention.

We report a case of a giant pancreatic pseudocyst in a 56-year-old-female with a history of heavy alcohol use presenting with shortness of breath, general malaise, and dyspnea on exertion. Initial computed tomography (CT) scan demonstrated a giant pancreatic pseudocyst measuring up to 22 cm in the largest diameter. The patient was hospitalized, and an endoscopic cystogastrostomy was performed. Once the patient was stabilized, the cystogastrostomy stent was removed and replaced with a pigtail catheter. CT scan at three-month follow-up demonstrated no evidence of fluid re-accumulation.

Due to the large size of giant pancreatic pseudocysts, drainage of the pseudocyst is the most appropriate treatment. There are different treatment modalities to achieve the goal of draining pseudocysts. One of the most commonly used treatments is an endoscopic ultrasound-guided cystogastrostomy, which this case highlights as an acceptable treatment option for giant pancreatic pseudocyst.

## Introduction

A pancreatic pseudocyst is a collection of pancreatic fluid encapsulated within a well-defined wall in the peripancreatic tissues [1]. The incidence of pancreatic pseudocyst development was described in one study as 14.6% in patients with acute pancreatitis and 41.8% in cases of acute-on-chronic pancreatitis [2]. This condition results from complications of acute or chronic pancreatitis, which is most often due to chronic alcoholism, and does not develop spontaneously without pancreatitis. Chronic pseudocysts present for over eight weeks are unlikely to dissipate spontaneously [3]. The management of pancreatic pseudocyst is largely determined by the etiology, size, and patient status at the time of diagnosis. The main options for the treatment of persistent pancreatic pseudocyst are drainage by either percutaneous, endoscopic, or open approach [4].

The term “giant” pancreatic pseudocyst is defined in literature as pseudocyst measuring >10 cm in diameter [4]. Giant pancreatic pseudocysts are a rare occurrence, as investigations into literature show that there have only been a small handful of cases of “giant” pancreatic pseudocyst reported [5-11]. This is a case presentation of a giant pancreatic pseudocyst secondary to chronic alcoholism, including the treatment by way of endoscopic ultrasound-guided cystogastrostomy.

## Case presentation

**Figure 1:**
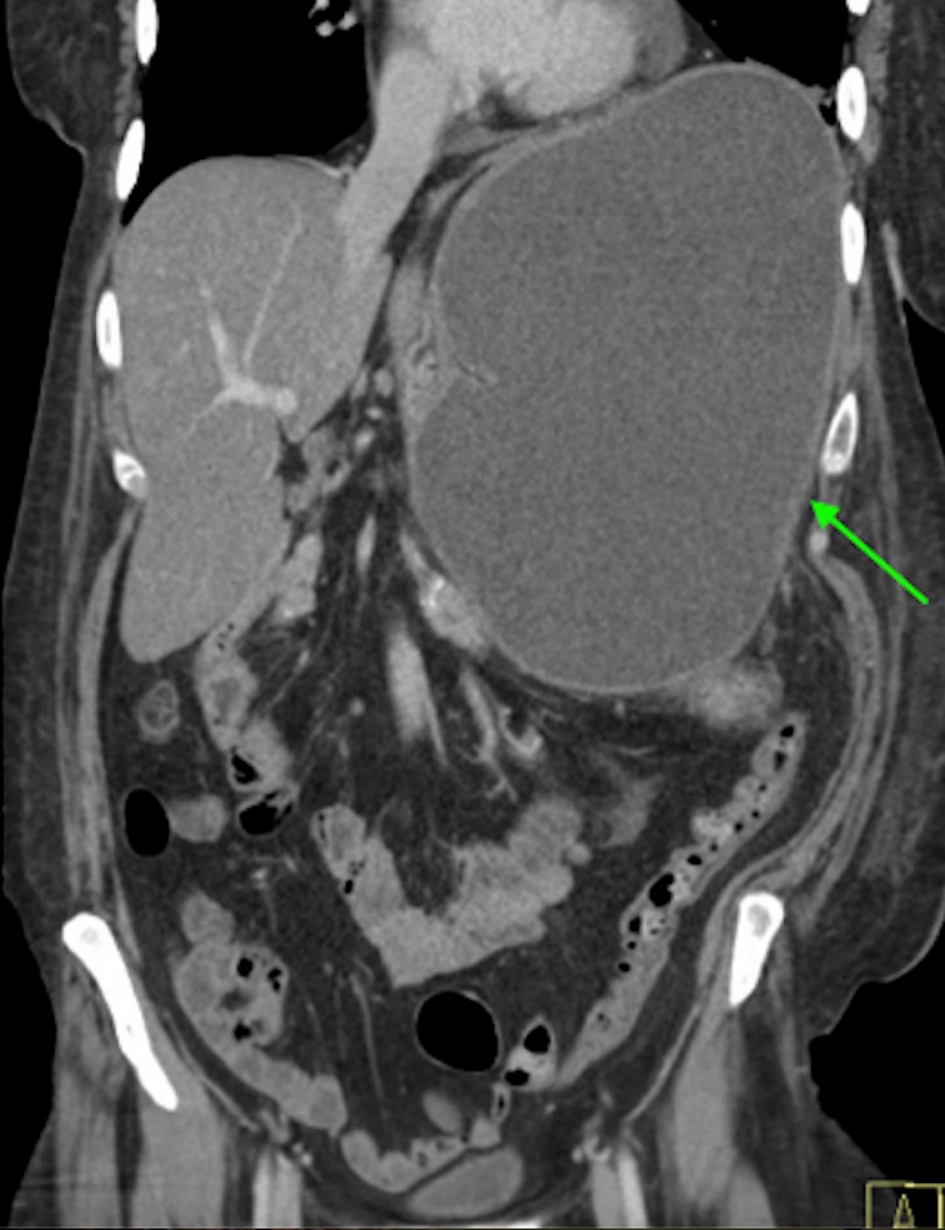
Coronal view CT of the abdomen and pelvis with intravenous contrast demonstrating a large fluid collection in the left upper quadrant (arrow). CT - computed tomography

**Figure 2:**
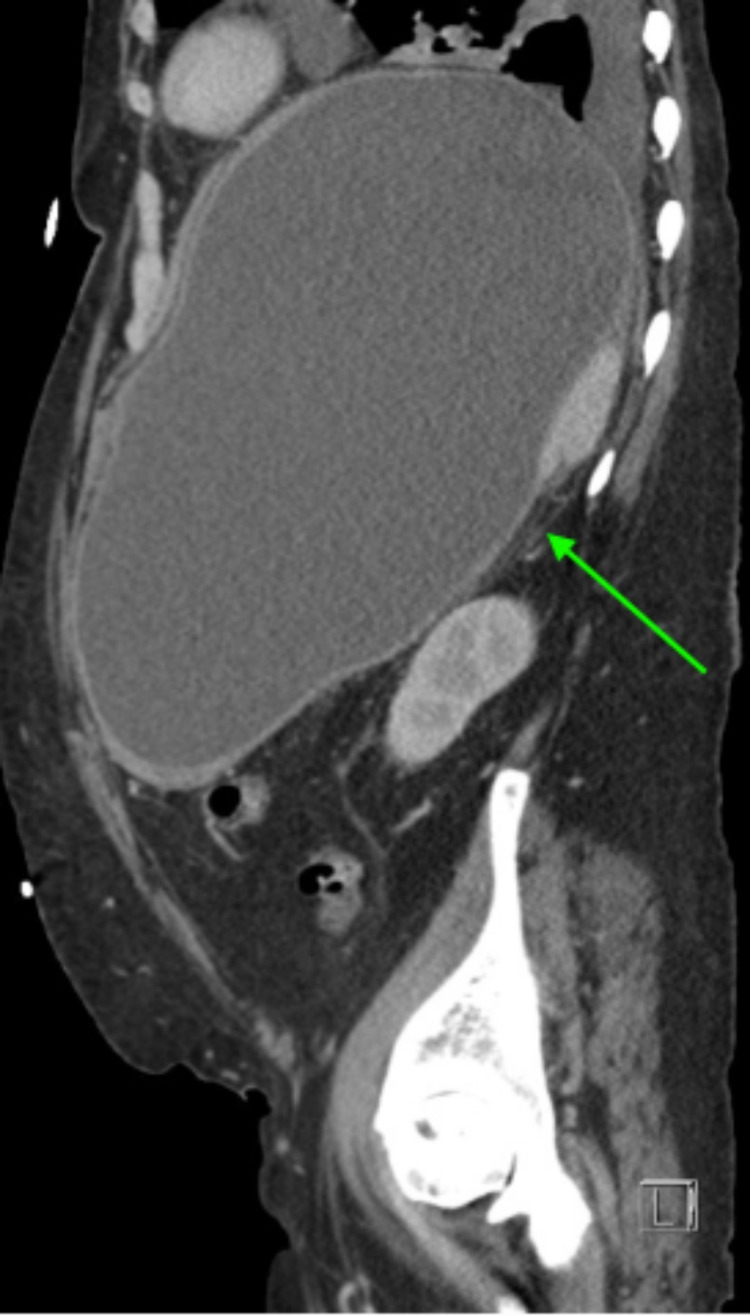
Sagittal view CT of the abdomen and pelvis with intravenous contrast demonstrating a large fluid collection in the left upper quadrant (arrow). CT - computed tomography

We present the case of a 56-year-old female with a past medical history pertinent for depression, cirrhosis, chronic pancreatitis, tobacco abuse, and alcohol abuse. She presented to the emergency department (ED) for shortness of breath, general malaise, and dyspnea on exertion after a mechanical fall while ambulating and subsequent motor vehicle collision earlier in the day. The patient also endorsed several months of progressive loss of appetite and increasing nausea with vomiting over several days prior to evaluation. Alcohol use was significant for consuming about six beers plus wine or liquor daily. The examination findings were significant for profound abdominal distention and palpation of a firm mass in the left upper quadrant. CT of the abdomen and pelvis revealed a “large developing fluid collection in the left upper quadrant of the abdomen estimated up to 22 cm maximal dimension with regional mass effect.” With a history of chronic pancreatitis and atrophy of the pancreas with calcifications present on the imaging obtained, a pancreatic pseudocyst was the most likely differential (Figures 1-2).

**Figure 3:**
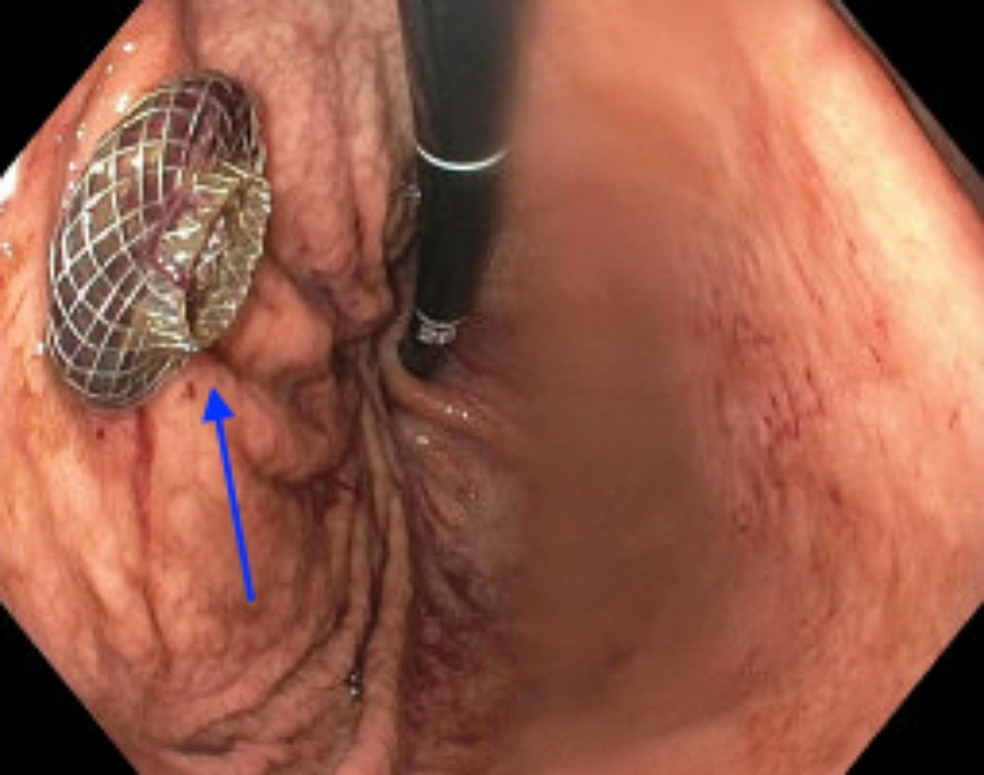
Endoscopic view of the stomach with cystogastrostomy stent in place (arrow).

**Figure 4:**
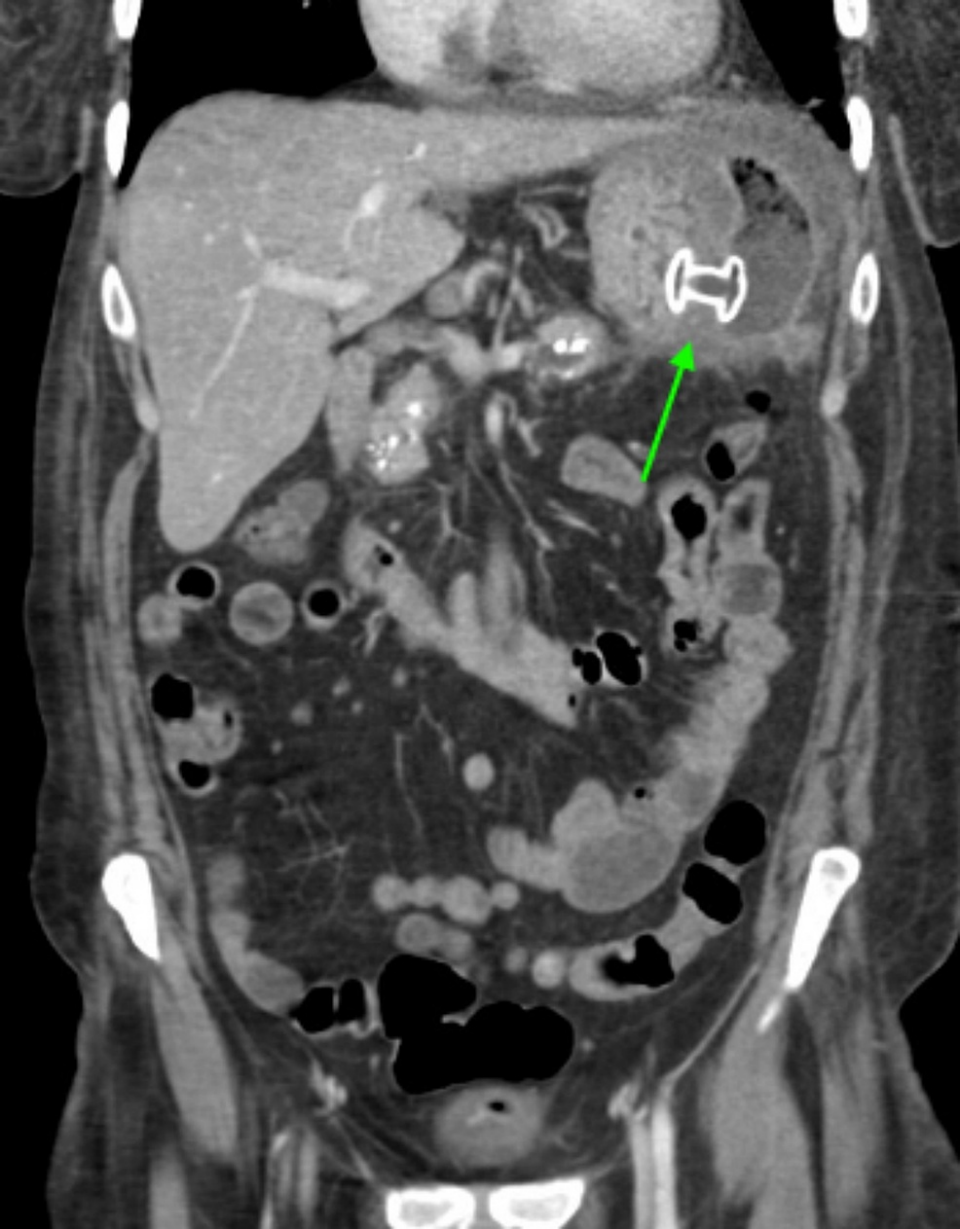
Coronal view CT of the abdomen and pelvis with intravenous contrast post cystogastrostomy stent (arrow) placement demonstrating decompression of the giant pseudocyst. Residual gas and fluid are present in the collection. CT - computed tomography

**Figure 5:**
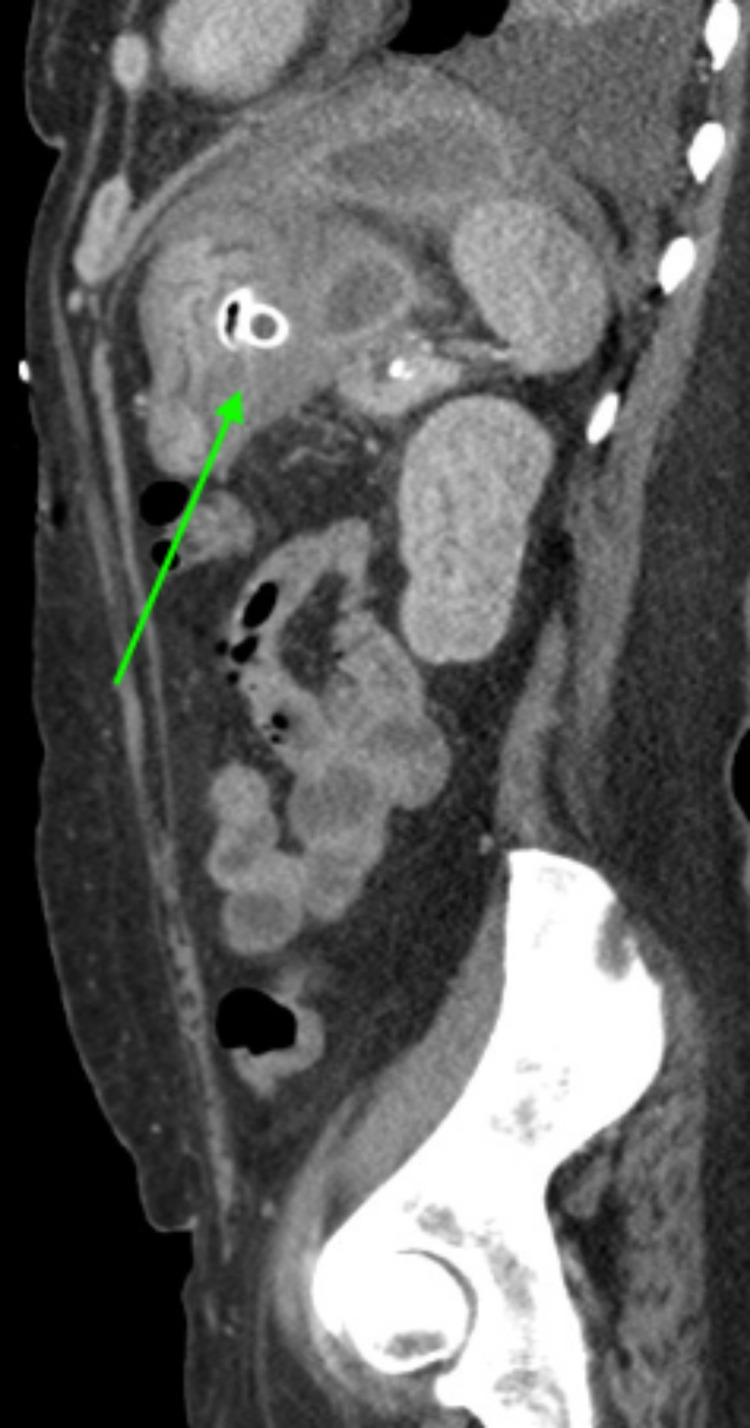
Sagittal view CT of the abdomen and pelvis with intravenous contrast post cystogastrostomy stent (arrow) placement demonstrating decompression of the giant pseudocyst. Residual gas and fluid are present in the collection. CT - computed tomography

**Figure 6:**
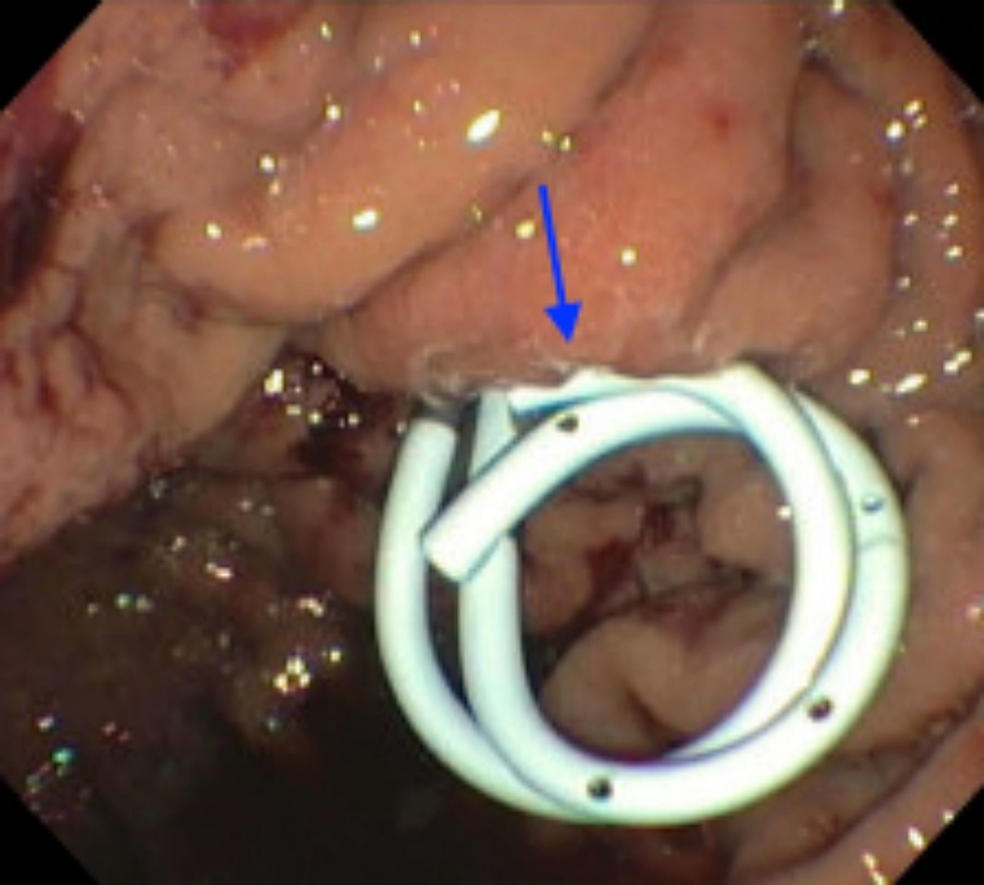
Endoscopic view of the stomach after the cystogastrostomy stent was exchanged for double pigtail catheters (arrow) connecting the pancreatic pseudocyst to the stomach.

Interventional gastroenterology was appropriately consulted with recommendations to correct electrolyte derangements, notable for hyponatremia to 125, prior to endoscopic intervention. Following medical optimization for the presenting condition, the patient underwent a successful endoscopic ultrasound-guided pancreatic pseudocyst cystogastrostomy. Procedural endosonography revealed a 21 × 12 cm in maximal cross-sectional diameter large, anechoic lesion consistent with suspected pseudocyst in the peripancreatic region. Intervention by the consulted specialists resulted in a cystogastrostomy with stent placement and subsequent drainage of 4.5 L of dark, debris-laden fluid with additional drainage into the gastric lumen (Figures 3-5). The stent was exchanged for double pigtail catheters connecting the pseudocyst to the stomach prior to the patient’s discharge (Figure 6).

**Figure 7:**
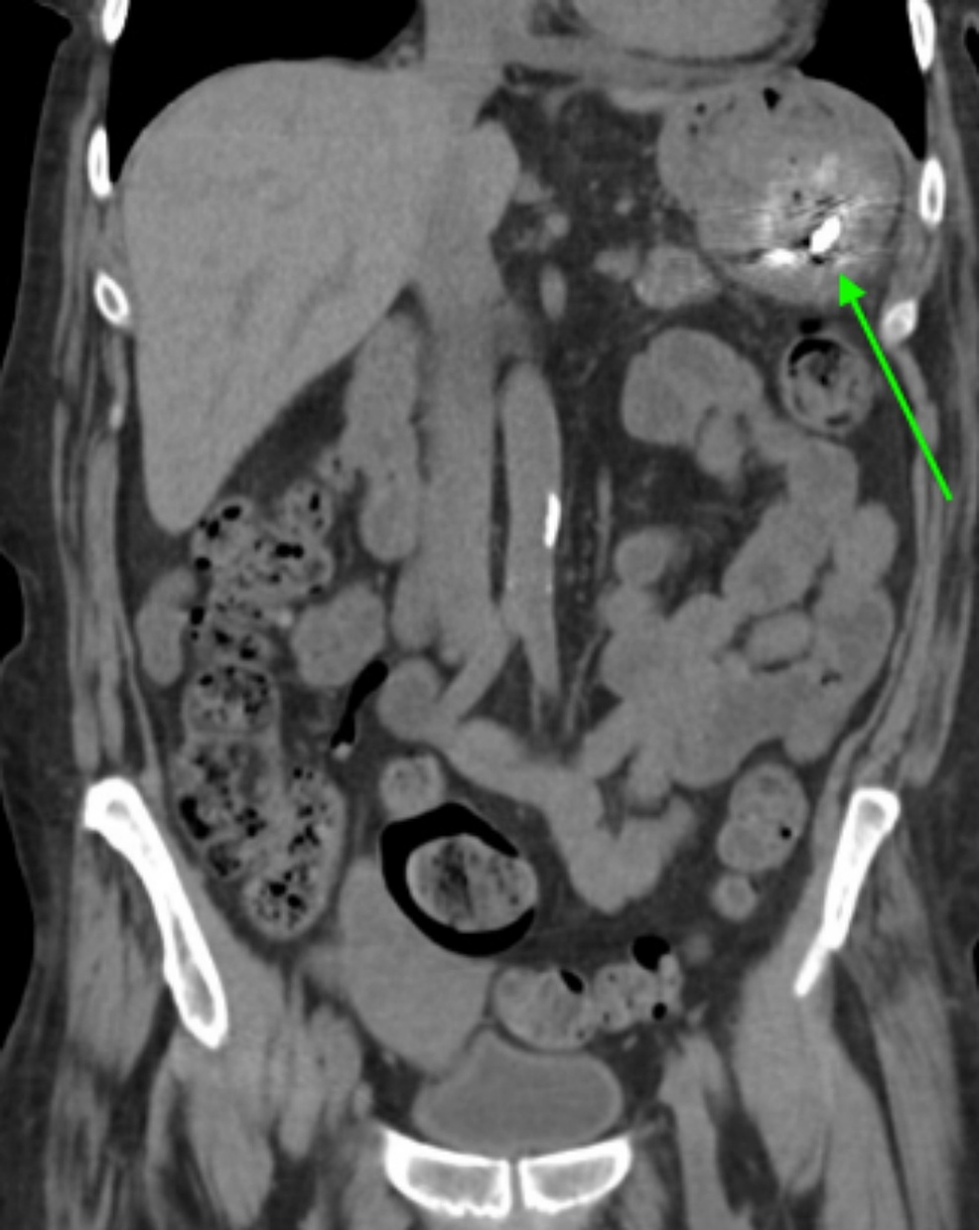
Coronal view CT of the abdomen and pelvis with intravenous contrast at three-month follow-up showing a drainage catheter (arrow) extending from the stomach to the collapsed pseudocyst within the left upper quadrant. There was no evidence of fluid re-accumulation. CT - computed tomography

**Figure 8:**
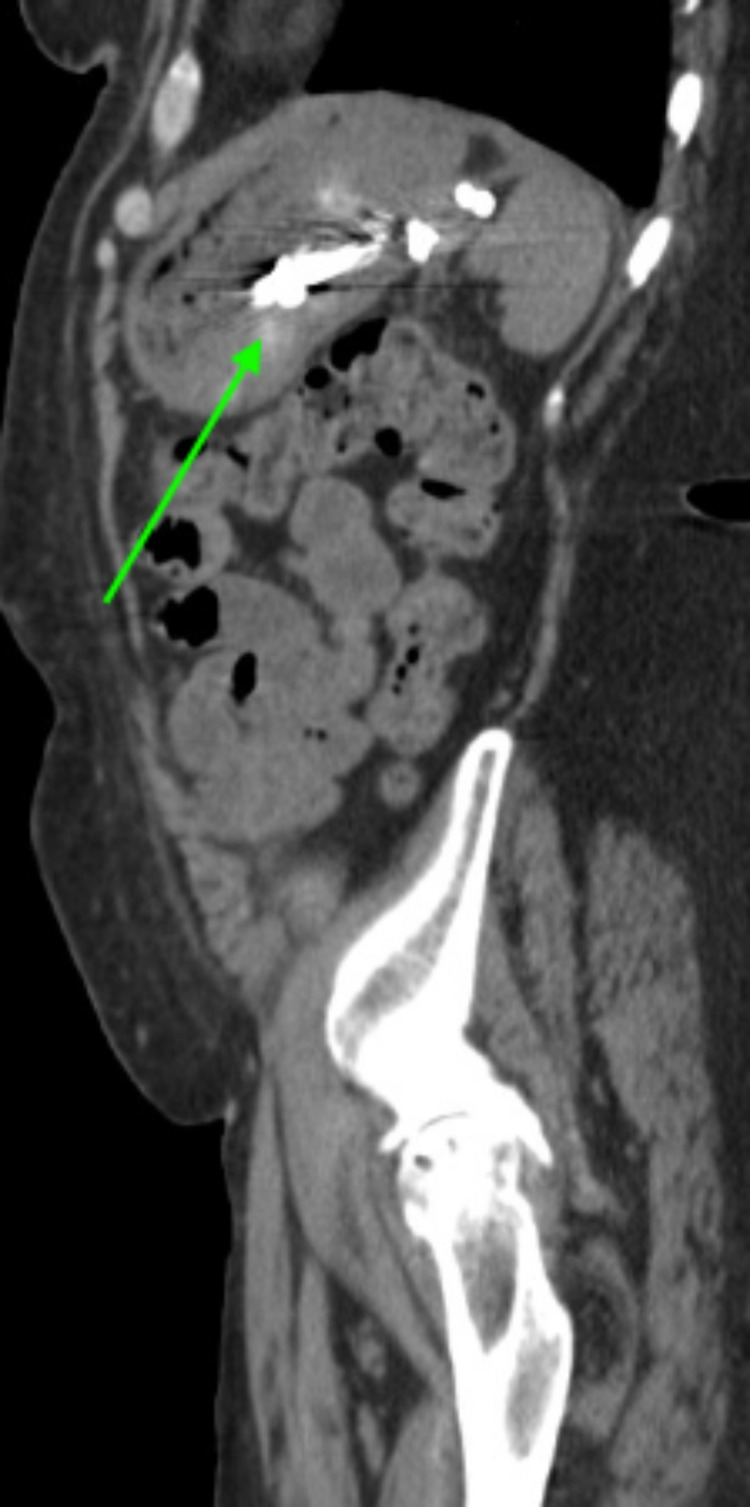
Sagittal view CT of the abdomen and pelvis with intravenous contrast at three-month follow-up showing a drainage catheter extending from the stomach to the collapsed pseudocyst within the left upper quadrant. There was no evidence of fluid re-accumulation. CT - computed tomography

The patient was seen once again approximately three months after her initial discharge. Imaging at that time showed that there was no evidence of re-accumulation in the pseudocyst (Figures 7-8).

## Discussion

To be considered a pancreatic pseudocyst, a peripancreatic fluid collection must be at least four weeks old with a cyst wall present. Drainage of a mature pseudocyst is the treatment of choice, which can be performed by excision, external drainage, internal drainage, or endoscopic drainage. It has been reported that there is not a significant difference in the resolution of the pseudocyst with surgical versus endoscopic techniques; thus, the minimally invasive cystogastrostomy was recommended [12]. A giant pancreatic pseudocyst of the size reported in this case has rarely been seen; as from our literature review, it is one of the 10 largest reported, and management strategies are varied for pseudocysts of this size. Procedures for the management of giant pancreatic pseudocyst include external drainage, video-assisted pancreatic necrosectomy, laparoscopic and open cystogastrostomy, and endoscopic ultrasound-guided internal drainage. Some complications of cystogastrostomy included incomplete drainage and death. However, most recent case reports for pancreatic pseudocyst of this size had favorable outcomes when open cystogastrostomy or endoscopic cystogastrostomy were performed [5,6].

Cystogastrostomy can be performed via the open or endoscopic method and uses a stent to connect the pseudocyst cavity to the stomach. A recent study showed that endoscopic cystogastrostomy had similar outcomes to surgical drainage in acute pancreatic pseudocyst. The most common reason for the failure of the endoscopic method was necrosis, but stent migration, inadequate opening size, and the presence of multiple locations within the cyst were also reported. Endoscopic drainage can lead to a shorter recovery time and hospital stay compared to surgery, but this is not always the case [13].

There is no established standard of care for the management of giant pancreatic pseudocysts due to the rarity of the condition. In our case, endoscopic ultrasound-guided cystogastrostomy was performed with later removal of the stent and placement of the pigtail catheter for continued drainage. The patient had a prolonged hospital stay due to a variety of factors including altered mental status from alcohol withdrawal. Ultimately, the patient fully recovered during the hospital course. Due to multiple comorbidities, she has presented to the ED multiple times since her procedure for complaints such as generalized weakness, hypotension, and extremity edema. Upon most recent follow-up imaging, approximately three months after the initial procedure, the collapse of the pseudocyst was noted with no fluid re-accumulation. There have been no complications related to the cystogastrostomy or pigtail catheter placement noted, and routine primary care follow-up has been reported.

Thus, we contend that endoscopic ultrasound-guided cystogastrostomy can be successfully performed for drainage of the giant pancreatic pseudocyst. In our case, although the patient had other comorbidities, there were no complications related to either procedure reported, and resolution of the pseudocyst without recurrence was sustained. A pancreatic pseudocyst of this size may require repeated procedures to ensure proper drainage and should be followed closely. This case demonstrates that a minimally invasive approach can successfully manage a giant pancreatic pseudocyst.

## Conclusions

Giant pancreatic pseudocyst is a rare but serious sequela of pancreatitis, and it is infrequently reported in the currently available literature. This case demonstrates that endoscopic ultrasound-guided cystogastrostomy is a viable treatment option for giant pancreatic pseudocyst. This procedure is minimally invasive and can allow for sustained resolution of the pseudocyst. An immediate success, in this case, was seen by decompression of the pseudocyst on imaging shortly after the initial procedure, and at three months, there was no re-accumulation within the pseudocyst. The procedure in our case demonstrated that this is a safe treatment as well, as the patient had no complications directly related to the procedure despite multiple other comorbidities. Cystogastrostomy may require frequent follow-up and repeat endoscopy to ensure that proper drainage takes place, but this is a method that can allow for the resolution of giant pancreatic pseudocyst in the least invasive manner possible.
